# The Unilateralist’s Curse and the Case for a Principle of Conformity

**DOI:** 10.1080/02691728.2015.1108373

**Published:** 2016-01-26

**Authors:** Nick Bostrom, Thomas Douglas, Anders Sandberg

**Keywords:** The Winner’s Curse, Disagreement, Rationality, Aumann

## Abstract

In some situations a number of agents each have the ability to undertake an initiative that would have significant effects on the others. Suppose that each of these agents is purely motivated by an altruistic concern for the common good. We show that if each agent acts on her own personal judgment as to whether the initiative should be undertaken, then the initiative will be undertaken more often than is optimal. We suggest that this phenomenon, which we call *the unilateralist’s curse*, arises in many contexts, including some that are important for public policy. To lift the curse, we propose a *principle of conformity*, which would discourage unilateralist action. We consider three different models for how this principle could be implemented, and respond to an objection that could be raised against it.

## 1. Introduction

Consider the following hypothetical scenarios:

(1) A group of scientists working on the development of an HIV vaccine has accidentally created an air-transmissible variant of HIV. The scientists must decide whether to publish their discovery, knowing that it might be used to create a devastating biological weapon, but also that it could help those who hope to develop defenses against such weapons. Most members of the group think publication is too risky, but one disagrees. He mentions the discovery at a conference, and soon the details are widely known.
(2) A sports team is planning a surprise birthday party for its coach. One of the players decides that it would be more fun to tell the coach in advance about the planned event. Although the other players think it would be better to keep it a surprise, the unilateralist lets word slip about the preparations underway.
(3) Geoengineering techniques have developed to the point that it is possible for any of the world’s twenty most technologically advanced nations to substantially reduce the earth’s average temperature by emitting sulfate aerosols. Each of these nations separately considers whether to release such aerosols. Nineteen decide against, but one nation estimates that the benefits of lowering temperature would exceed the costs. It presses ahead with its sulfate aerosol program and the global average temperature drops by almost 1°.

In each of these cases, each of a number of agents is in a position to undertake an initiative, *X*. Suppose that each agent decides whether or not to undertake *X* on the basis of her own independent judgment of the value of *X*, where the value of *X* is assumed to be independent of *who* undertakes *X*, and is supposed to be determined by the contribution of *X* to the common good.^1^ Each agent’s judgment is subject to error—some agents might overestimate the value of *X*, others might underestimate it. If the true value of *X* is negative, then the larger the number of agents, the greater the chances that at least one agent will overestimate *X* sufficiently to make the value of *X* seem positive. Thus, if agents act unilaterally, the initiative is too likely to be undertaken, and if such scenarios repeat, an excessively large number of initiatives are likely to be undertaken. We shall call this phenomenon the *unilateralist’s curse*.

Though we have chosen to introduce the unilateralist’s curse with hypothetical examples, it is not merely a hypothetical problem. There are numerous historical examples, ranging from the mundane to the high-tech. Here is one:

Until the late 1970s, the mechanism of the hydrogen bomb was one of the world’s best kept scientific secrets: it is thought that only four governments were in possession of it, each having decided not to divulge it. But staff at the Progressive magazine believed that nuclear secrecy was fuelling the Cold War by enabling nuclear policy to be determined by a security elite without proper public scrutiny. They pieced together the mechanism of the bomb and published it in their magazine, arguing that the cost, in the form of aiding countries such as India, Pakistan and South Africa in acquiring hydrogen bombs, was outweighed by the benefits of undermining nuclear secrecy.^2^

Another possible example from atomic physics had occurred several decades earlier:

In 1939 the Polish nuclear physicist Joseph Rotblat noticed that the fission of uranium released more neutrons than used to trigger it, realizing that it could produce a chain reaction leading to an explosion of unprecedented power. He assumed that other scientists elsewhere were doing similar experiments, and were thus in a position to release similar information, an assumption that turned out to be correct. Initially, Rotblat vowed to tell no-one of his discovery, believing it to be a threat to mankind, and it is plausible that others did likewise, for similar reasons. However, when the war broke out, Rotblat decided that releasing the information was now in the public interest, given the likelihood that the Germans were working on an atomic bomb. He confided in colleagues and thus unilaterally triggered the United Kingdom’s atomic bomb project.^3^

Rotblat was later to leave the Manhattan Project, coming to the view that his had overestimated the German nuclear threat, and underestimated the likelihood that the US would use an atomic bomb offensively.

It is perhaps too soon to say whether these unilateral actions were suboptimal. But in other cases, it is clearer that unilateral action led to a suboptimal outcome:

In the mid-nineteenth century there were virtually no wild rabbits in Australia, though many were in a position to introduce them. In 1859, Thomas Austin, a wealthy grazier, took it upon himself to do so. He had a dozen or two European rabbits imported from England and is reported to have said that “The introduction of a few rabbits could do little harm and might provide a touch of home, in addition to a spot of hunting.”^4^ However, the rabbit population grew dramatically, and rabbits quickly became Australia’s most reviled pests, destroying large swathes of agricultural land.^5^

The abovementioned examples were isolated incidents, but similar situations occur regularly in some spheres of activity, for instance, in the media:

Media outlets sometimes find themselves in the situation that journalists have access to information that is of public interest but could also harm specific individuals or institutions: the name of a not-yet charged murder suspect (publication may bias legal proceedings), the news that a celebrity committed suicide (publication may risk copycat suicides), or sensitive government documents such as those leaked by Wikileaks and Edward Snowden (publication may endanger national security). It is enough that one outlet decides that the public interest outweighs the risk for the information to be released. Thus, the more journalists have access to the information the more likely it is to be published.

Unilateralist situations also regularly crop up in regards to new biotechnologies:

Gene drives, a technique for inducing altered genes to be inherited by nearly all offspring (rather than just 50%) of a genetically modified organism, have potential for spreading altered genes across a population, enabling ecological control (e.g. making mosquitos incapable of spreading malaria or reducing herbicide resistance) but also potentially creating worrisome risks (e.g. to genetic diversity or of sabotage). Here unilateral action could both be taken in releasing a particular altered organism into the environment, and in releasing the information about how to produce it in the first place. There is scientific disagreement on the utility and risk of both.^6^

## 2. The Unilateralist’s Curse: A Model

The unilateralist’s curse is closely related to a problem in auction theory known as the winner’s curse. The winner’s curse is the phenomenon that the winning bid in an auction has a high likelihood of being higher than the actual value of the good sold.^7^ Each bidder makes an independent estimate and the bidder with the highest estimate outbids the others. But if the average estimate is likely to be an accurate estimate of the value, then the winner overpays. The larger the number of bidders, the more likely it is that at least one of them has overestimated the value.

The unilateralist’s curse and the winner’s curse have the same basic structure. The difference between them lies in the goals of the agents and the nature of the decision. In the winner’s curse, each agent aims to make a purchase if and only if doing so will be valuable *for her*. In the unilateralist’s curse, the decision-maker chooses whether to undertake an initiative with an eye to the common good, that is, seeking to undertake the initiative if and only if the initiative contributes positively to the common good.

The unilateralist’s curse can be illustrated using a simple mathematical model. Assume *N* agents, each considering whether to undertake an initiative. Each agent wishes to proceed if and only if the value of the initiative is positive, but the agents do not know the true value *V* ^*^ of the initiative (which may be negative or positive). Instead each agent forms an estimate that is the sum of *V* ^*^ and a random independent error *d* drawn from a distribution with cumulative distribution function *F*(*d*)*.* This means that the probability *p* that any given agent will estimate the value of the initiative to be positive when it is in fact negative (*V* ^*^ < 0) is *p* = 1 − *F*(−*V* ^*^).^8^ The probability *P* that at least one of the agents will incorrectly estimate the value to be positive is *p* = 1 − (1 − *p*)^*N*^ *=* 1 − *F*(−*V* ^*^)^*N*^.

**Figure 1:**
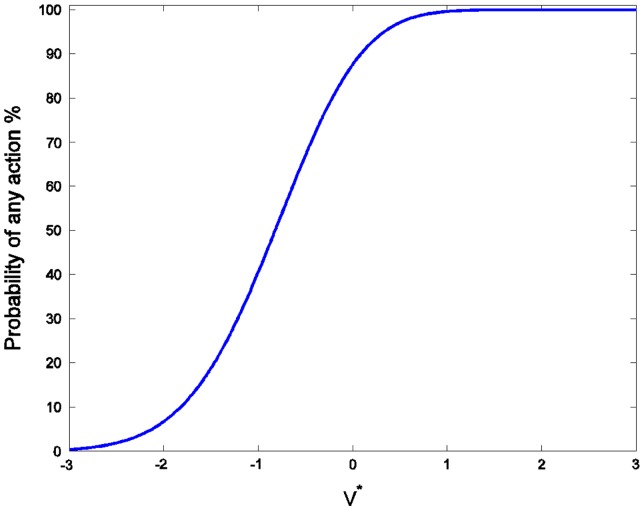
The probability of an initiative being undertaken as a function of the actual value, *V* ^*^, for five agents and assuming normally distributed errors with variance 1 (these assumptions will be used in all subsequent figures except when otherwise noted). Note that 50% probability of action occurs near a value of −1: a strong unilateralist bias exists.

For the case with 5 agents and *d* as a random error drawn from a normal distribution with standard deviation 1 and mean zero, the probability that any initiative will be undertaken (regardless of whether it is a good idea or not) is high even when the true value is quite negative, and the probability rises steeply as the true value of the initiative approaches zero from below (Figure 1).

**Figure 2:**
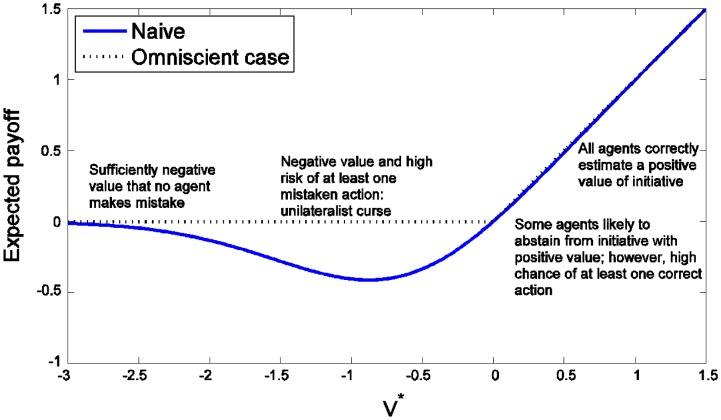
The expected payoff for naive agents (who act if and only if their evaluation of the initiative is positive) and ideal omniscient estimators who are assumed to know the true value.

For mildly negative values of the initiative there is nearly always someone who misjudges the value of the initiative and undertakes it. There is no problem for positive initiatives since even if one or two agents are overly cautious, it is very likely that somebody will undertake the initiative, which is the optimal result (Figure 2).

**Figure 3:**
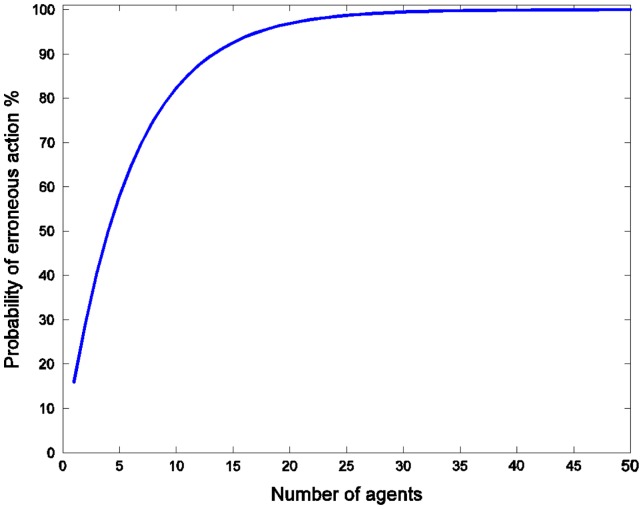
Probability of an erroneous action in the case of *V* ^*^ = −1 for different numbers of agents.

Increasing the number of agents capable of undertaking the initiative also exacerbates the problem: as *N* grows, the likelihood of someone proceeding incorrectly increases monotonically towards 1.^9^ The magnitude of this effect can be quite large even for a relatively small number of agents. For example, with the same error assumptions as above, if the true value of the initiative *V* ^*^ = −1 (the initiative is undesirable), then the probability of erroneously undertaking the initiative grows rapidly with *N*, passing 50% for just four agents (Figure 3).

There are six features of the unilateralist’s curse that that need to be emphasized.

First, in cases where the curse arises, the risk of erroneously undertaking an initiative is not caused by self-interest. In the model, all agents act for the common good, they simply disagree about the contribution of the initiative to the common good.^10^

Second, though the curse could be described as a group-level bias in favor of undertaking initiatives, in does not arise from biases in the individual estimates of the value that would result from undertaking the initiative. The model above assumes symmetric random errors in the estimates of the true value.^11^

Third, there is a sense in which the unilateralist’s curse is the obverse of Condorcet’s jury theorem.^12^ The jury theorem states that the *average* estimate of a group of people with above 50% likelihood of guessing correctly and with uncorrelated errors will tend to be close to the correct value, and will tend to move closer to the true value as the size of the group increases. But what is also true, and relevant to the argument in this paper, is that the *highest* estimate will tend to be above the true value, and the expected overestimation of this highest estimate *increases* with the size of the group. In the cases we are interested in here, it is the highest estimate that will determine whether an initiative is undertaken, not the average estimate.

Fourth, though we have chosen to illustrate the curse using initiatives that are (probably) irreversible, the problem can arise in other cases too. The problem becomes sharper if the initiative is irreversible, but even for actions that can be undone the problem remains in a milder form. Resources will be wasted on undoing erroneous initiatives, and if the bad consequences are not obvious they might occur before the problem is noticed. There might even be a costly tug-o-war between disagreeing agents.

Finally, fifth, though we have thus far focused on cases where a number of agents can undertake an initiative and it matters only whether at least one of them does so, a similar problem arises when any one of a group of agents can *spoil* an initiative—for instance, where universal action is required to bring about an intended outcome. Consider the following example:

In Norse mythology, the goddess Hel of the underworld promised to release the universally beloved god Baldr if all objects, alive and dead, would shed a tear for him. All did, except the giantess Þökk. The god was forced to remain in the underworld.^13^

Similar situations can arise when all the actors in a play must come together in order for a rehearsal to take place, when all members of committee must attend a meeting in order for it to be quorate, or when all signatories to an international treaty must ratify it in order for it to come into effect. The United Nations Security Council frequently provides examples of unilateral spoiling. The five permanent members of the Council—currently China, France, Russia, the United Kingdom and the United States—each possesses the power to veto the adoption of any non-procedural resolution. In the early years of the Council, this veto power was frequently employed by the Soviet Union to block applications for new membership of the United Nations. More recently, it has been used by the United States to block resolutions criticizing Israel, and by Russia and China to block resolutions on the Syria conflict.^14^ While some of these vetoes presumably reflect differences in the national interests of the council members, others may reflect different estimations of the contribution that a resolution would make to the common good. Certainly, considerations relating to the common good are often invoked in their defence. For instance, the United States’ 2011 veto of a draft resolution condemning Israeli settlements in Palestinian territory was defended on the grounds that the resolution would be an impediment to peace talks.^15^

These cases of unilateral spoiling or abstinence are formally equivalent to the original unilateralist curse, with merely the sign reversed.

Since the problem in these cases is the result of *unilateral* abstinence, it seems appropriate to include them within the scope of the unilateralist’s curse. Thus, in what follows, we assume that the unilateralist’s curse can arise when each member of a group can unilaterally undertake *or spoil* an initiative (though for ease of exposition we sometimes mention only the former case).

## 3. Lifting the Curse

Let a unilateralist situation be one in which each member of a group of agents can undertake or spoil an initiative regardless of the cooperation or opposition of other members of the group. We will say that a policy would lift the unilateralist’s curse if universal adherence to it by all agents in unilateralist situations should be expected (*ex ante*) to eliminate any surfeit or deficit of initiatives that the unilateralist’s curse might otherwise produce.

*The Principle of Conformity*

When acting out of concern for the common good in a unilateralist situation, reduce your likelihood of unilaterally undertaking or spoiling the initiative to a level that *ex ante* would be expected to lift the curse.

In the following subsections we will explore various ways in which one might bring oneself into compliance with this principle.^16^ These can be organized around three models: collective deliberation, epistemic deference, and moral deference. The three models are applicable in somewhat different circumstances, and their suitability might depend on the type of agents involved.

It should be noted that, though some of the methods discussed below do not require agents to be aware of the nature of the situation, most hinge on agents recognizing that they are in an unilateralist situation. However, this is not to say that agents must be able to identify the other parties to the unilateralist situation: this is necessary for some but not all of our proposed solutions.

### 3.1. The Collective Deliberation Model

A first line of defense against the unilateralist’s curse could be to share data and reasoning between agents in the hope that this will resolve their disagreement about the desirability of proceeding with the contested initiative.

In some cases, however, extensive information sharing among all potential decision-making agents is impractical. Communication is often costly and time-consuming, and participants in a unilateralist situation may not be able to identify one another. Furthermore, in certain cases information disclosure might itself be the initiative whose desirability is in dispute, such as when information hazards are associated with disseminating relevant data.^17^

Moreover, even when information is fully shared, a consensus can remain elusive. Disagreements about the net value of undertaking some project often persist after decision-makers have been thoroughly briefed on all obviously relevant and easily communicable facts and after having had opportunities to engage in joint deliberation.

Because complete information sharing may not be practical or desirable, and because it may not produce consensus when it does occur, the principle of conformity requires us to explore additional models for lifting the unilateralist’s curse.

### 3.2. The Meta-rationality Model

One approach would be to appeal to each agent’s reflective rationality. A party to an epistemic disagreement should ideally reflect on the fallibility of their own judgment and adjust their posterior probability to take into account the fact that other agents have different opinions.

Robert Aumann has shown that rational Bayesian agents with identical priors and common knowledge of each other’s posteriors (and of each other’s rationality) must have identical posterior probabilities.^18^ Disagreement between such agents is impossible. This sounds like good news: if all agents make the same estimate of the benefits of action, the unilateralist curse is lifted.

There is, however, some skepticism about the relevance of Aumann’s result for practical cases of disagreement.^19^ The assumption of identical priors, in particular, is problematic.^20^ Furthermore, the same challenges that can make data sharing difficult can also make it difficult to make each agent’s honest posterior probability estimates of the value of the initiative common knowledge among all agents.

It turns out, however, that sufficiently rational agents can manage the curse even without communication. In the literature on the winner’s curse it has been argued that rational expected utility-maximizing will not be affected by it.^21^ Rational agents will take the winner’s curse into account and adjust their bids accordingly. This is known as *bid shading*. Rational agents place bids that are lower than their *ex ante* expectation of the value of the good, but equal to their expectation of the value of the good conditional upon them winning the auction.

The counterpart of this response would be for agents in a unilateralist situation to estimate the value of the initiative conditional on the agent’s first-order estimate of the initiative’s value being the highest (or, in spoiler cases, the lowest).

In other words, on finding themselves in a unilateralist situation, each rational agent will initially estimate the value of the initiative based on his prior probability distribution. He will then take into account the case where his decision is decisive. In the case where agents can unilaterally undertake an initiative, the agent will condition on the situation in which he is the most sanguine and everybody else thinks the action should not be done. (In spoiler cases, the agent conditions on the situation in which he is the most pessimistic and everybody else thinks the initiative should be undertaken.) He then creates a posterior distribution of value that is used to make an adjusted decision.
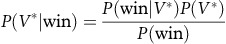



where “win” represents being the deciding agent. Note that this typically requires knowing or estimating the number of other agents.

*Example*

In the simple case where the agent assumes all other agents have the same priors and are acting independently, only differing in the noisy data about *V* ^*^ they have received:
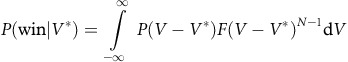



where *F*(*V*) is the cumulative distribution function of the errors. The posterior distribution of *V* ^*^ becomes:
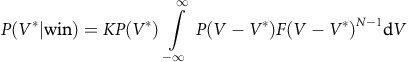



where *K* is a normalization constant. The posterior action should then be based on the expectation *E*(*V* ^*^ *|*win).

**Figure 4:**
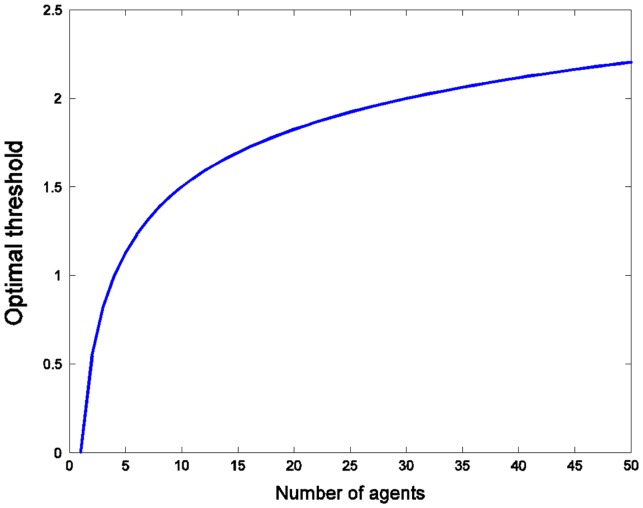
The optimal threshold *T*_opt_(*N*) for action as a function of the number of agents. Agents who only act if the perceived value of the initiative is higher than *T*_opt_(*N*) will maximize their expected (joint) result.

**Figure 5:**
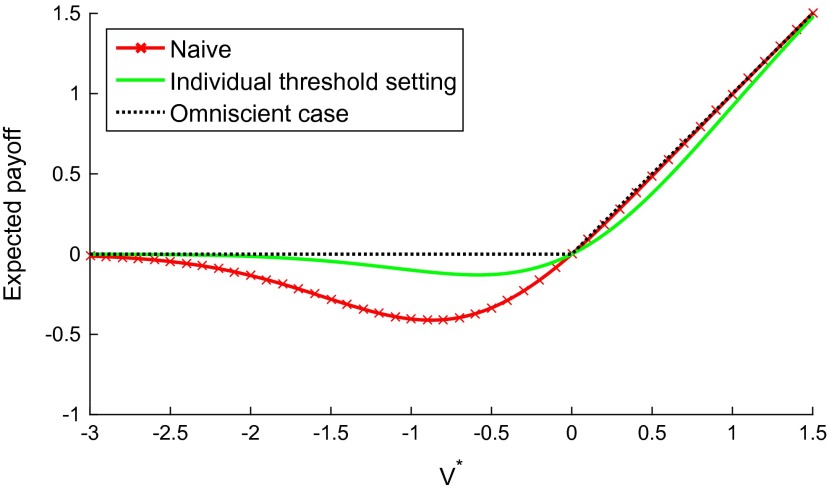
The expected payoff for different actual values of the initiative for alternative ways of handling the unilateralist’s curse. Using the optimal individual threshold *T*_opt_(5) reduces the losses significantly.

If the agents choose to act when the received data is above a fixed threshold *T*, *V* ^*^ is normally distributed with zero mean and variance 1, and they get estimates of *V* ^*^ with normal noise (again with mean zero and variance 1), then the optimal threshold is the one that maximizes the expected value (Figure 4):
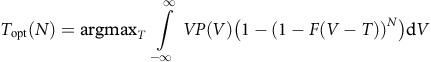



*T*_opt_(*N*) increases rapidly with *N*, reaching 0.54 for two agents and 1 for 4 agents: even for a small group it is rational to be far more cautious than in the single agent case. Note that in this case all agents are aware of the prior distribution, noise distribution, independence, and that the other agents are using this strategy (Figure 5).^22^

One might raise questions about the practical applicability of this sophisticated Bayesian approach, however. Even if rational Bayesian agents would agree, humans are at best approximations of rational Bayesian agents and they have far more limited mental computation power—even when leaving out biasing factors.^23^ Value in practical cases is also seldom in the form of easily manipulable and comparable scalar quantities. Hence implementing the sophisticated Bayesian approach to lifting the unilateralist’s curse might typically be infeasible.^24^

### 3.3. The Moral Deference Model

Suppose a unilateralist situation exists and that it is not feasible for all agents to lift the curse through communication and adjustment of beliefs. It might nevertheless be possible for the group to lift the curse if each agent complies with a moral norm which reduces the likelihood that he acts unilaterally, for example, by assigning decision-making authority to the group as a whole or to one individual within it. We call this the moral deference model.

In contrast to the two models presented above, the moral deference model does not require agents to defer to the group in forming their beliefs regarding the value of the initiative. However, it does require them to defer to the group in deciding whether to act on those beliefs. A slogan for this approach could be “comply in action, defy in thought.”

There are many norms such that universal compliance with the norm by a group of agents would lift the unilateralist’s curse. For example, a norm that assigned decision-making authority to an arbitrary member of the group would lift it. Consider the norm: when in a unilateralist situation, if you are the tallest person able to undertake the initiative, then undertake it if and only if you believe its value exceeds zero; if you are not the tallest person able to undertake the initiative, do not undertake it.

**Figure 6:**
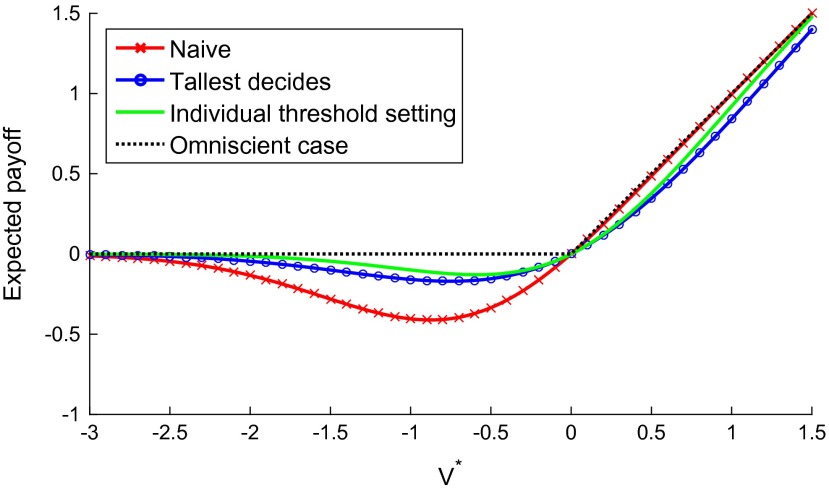
Expected payoff for different actual values of the initiative for alternative ways of handling the unilateralist curse. The tallest decides case achieves a significant reduction of loss, nearly reaching the payoff of the more complex Bayesian threshold method.

Universal compliance with this norm would prevent the unilateralist’s curse from arising in the sense that, in the absence of any bias towards or against action in the individual members of the group (and thus in the group’s tallest member), this norm will produce no group-level bias towards or against the initiative.^25^ The payoffs associated with this tallest-decides norm in a five-agent situation are depicted in Figure 6 below. The tallest-decides norm, however, has several epistemically and pragmatically unattractive features. For example, it does not protect against biases or errors that might impair the judgment of the group’s tallest member. Furthermore, it is very unlikely that such a norm would gain wide acceptance.

Fortunately, there are other norms that could lift the curse and may lack these unattractive features. One norm would recommend that agents conform to the rules of existing institutions that militate against unilateral action:

(1) When in a unilateralist’s situation, defer to existing institutions, such as laws or customs, if universal deference to those institutions would lift the unilateralist’s curse.

National and international laws often militate against the unilateralist’s curse, for example by specifying that decisions must be made democratically or by individuals or institutions that have been given special authority over a particular realm of decision-making. In other cases, there are informal conventions that may do the job. For example, following the publication early last decade of two studies thought by some to aid bioweapons development,^26^ a group of scientific journals agreed to introduce screening procedures to identify papers containing information that is especially prone to misuse and to seek external advice on the publication of such papers.^27^ Though these procedures lacked legal status, compliance with them by journals may have helped lift the curse.

One virtue of (1) is that, since it simply reinforces existing institutional norms which may already command significant support, it may be relatively easy for it to achieve wide acceptance. However, (1) will not lift the curse in all cases. In many areas with an international dimension, for example, there are no relevant international laws and deference to national laws would merely create a new unilateralist situation between nations: the nation that evaluates the initiative most positively is most likely to allow it. Moreover, (1) may sometimes recommend deferring to biased procedures or agents.

It might be possible for a group of agents to lift the curse even in cases where (1) fails by complying with a different norm, one that promotes the development of and compliance with a new procedure for group decision-making. This approach was adopted by a large group of American microbiologists in mid-1974 when they agreed to a moratorium on recombinant DNA research until such time as the safety concerns that it raised could be jointly discussed and resolved. The moratorium held until the now-famous Asilomar Conference, which took place in February 1975 and resulted in a broad-based agreement on guidelines regarding the conditions under which recombinant DNA research ought to proceed.^28^

The recombinant DNA moratorium and guidelines were developed via consensus, but another approach would be to employ a voting procedure. For example, suppose all agents faced with a unilateralist situation complied with the norm:

(2) When in a unilateralist’s situation, promote the holding of a majority vote among those capable of undertaking the initiative. If the vote takes place, then (a) defer to its verdict, and (b) encourage others to do likewise.

Universal compliance with this norm is likely to lift the curse. Since it is effectively using the median estimate it is robust to outliers. It will also tend to reduce systematic bias at the group level provided that individual biases are at least partially independent of one another.^29^ And since majority voting is a common and widely accepted method for group decision-making, this norm would have relatively good prospects of gaining wide acceptance.

Compliance with norms (1) and (2) will, however, lift the unilateralist’s curse only when a high degree of communication and coordination is possible. There are other norms whose universal adoption could lift the curse even in the absence of communication and coordination. Consider the norm:

(3) When in a unilateralist situation, bring about the outcome if and only if you judge that a majority vote among those capable of undertaking the initiative would yield a majority in favor of doing so.

Insofar as each individual capable of undertaking the initiative makes an accurate prediction of the views of all others, universal adoption of this norm will eliminate any group-level bias due to the unilateralist’s curse. Even if predictions of the views of others are inaccurate (e.g. because each agent overestimates the extent to which others share her views), universal adoption of this principle can still be expected to somewhat mitigate the unilateralist’s curse. It will tend to reduce the likelihood that those who value the initiative most favorably will undertake it, provided that these agents realize they are at the optimistic end of the spectrum.^30^

**Figure 7:**
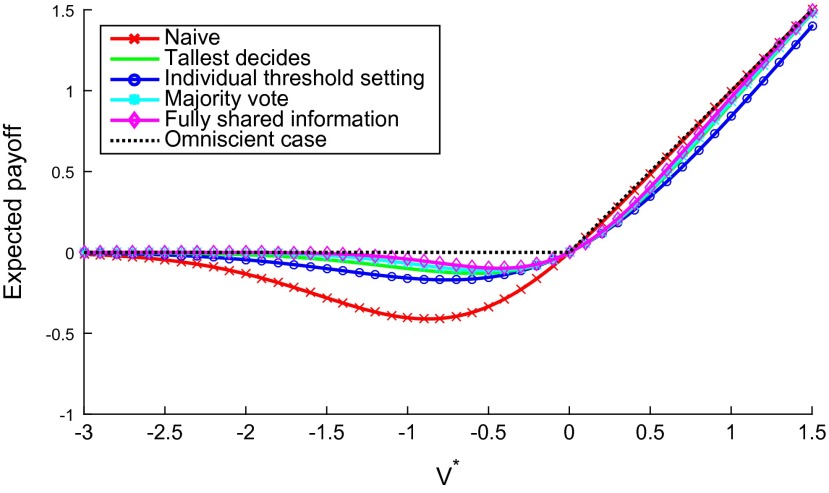
The expected payoff associated with universal compliance with six different strategies at different actual values of the initiative. The fully shared information strategy consists in pooling the information between the agents and acting on the group’s best joint estimate of *V* ^*^;^33^ this requires maximal communication. Despite the lack of communication in tallest decides and threshold setting, the agents achieve an average outcome close to the cases where communication is possible.

Figure 7 depicts, for a five-agent case, the expected payoffs associated with two of the norms discussed in this section—tallest decides, and the actual majority vote (norm (2))—and it compares these with other strategies described in Section 3.2 above. Under our assumptions, the majority vote does rather well—it is close to the maximum available payoff represented by the omniscient case.

However, in the real world, different strategies will work well in different cases. It is thus likely that the best norm to adopt, under the moral deference model, would be some composite of simple norms such as (1)–(3). For example, a group might adopt a norm that specifies that the group should act as specified by (1), (2) or (3) depending on what laws and conventions already exist, what forms of communication and coordination among group members are possible, and how costly such communication and coordination is likely to be, among other factors.

We do not wish to commit ourselves to norms (1)–(3) as the best building blocks from which to construct such a composite norm. We believe that each of (1)–(3) are at least plausible candidates for inclusion in a composite norm. However, there may be other norms that would more fully lift the curse or which have other advantages over (1)–(3). For example, there are well-known problems with majority voting which should perhaps lead us to prefer a different voting procedure under norms (2) and (3).

One other set of concerns regarding norms (2) and (3) warrants mentioning. Both of these norms involve holding a vote (real or hypothetical) *among agents capable of undertaking the initiative in question*. But it might be argued, on either epistemic or moral grounds, that any actual or hypothetical vote should include more individuals than merely those capable of undertaking the initiative. For example, perhaps the vote should include all whose capacity to evaluate the initiative passes some threshold of epistemic competence. Or perhaps, on moral grounds, the electorate should be expanded to include all individuals who will be affected by the initiative. Consider a case in which there are three agents who could undertake an initiative and two of the three judge that it would be best to do so. However, millions of others will be affected by the initiative and almost all of them judge that the initiative has net disvalue. In this case, it might seem morally preferable to hold (or imagine) a vote among all who will be affected by the initiative rather than limiting the vote to the three agent’s capable of undertaking it.

A more specific problem with excluding individuals who are incapable of undertaking the initiative is that this might seem to skew the vote. There might be some agents who are not capable of undertaking the initiative, but could have been capable of doing so; they are incapable only because they previously judged that undertaking the initiative would be a bad idea and thus ceased to develop the necessary capacities. Excluding these agents from a vote might seem to skew the vote in favor of those who deem the initiative to be valuable and who have thus sought to develop the capacities necessary to undertake it. Thus, limiting the vote to those capable of undertaking the initiative may be epistemically, as well as morally, problematic.

At the same time, it might be argued that some agents capable of undertaking the initiative should be *excluded* from the vote. Suppose that each of five nations is capable of undertaking some geoengineering project with worldwide consequences. Four agree to hold a majority vote among the five nations and to abide by the outcome of that vote. The fifth wishes to take part in the vote but is resolved to press ahead with the project regardless of the outcome of the vote. It might seem doubtful whether the first four nations should include the fifth in the vote. Arguably, deferring to a majority vote in unilateralist cases involves making a sacrifice. It involves giving away some of one’s autonomous decision-making authority. It might seem that it would be unfair for the fifth nation to exert an influence over the decisions of others by participating in a vote without also being prepared to make the same sacrifice that the others are prepared to make. This may count in favor of excluding the fifth nation. Excluding the fifth nation might also help to incentivize deference to majority votes in unilateralist situations.

There are thus arguments both for expanding and for restricting the group of agents given a vote in norms (2) and (3). We cannot assess these arguments here. We mention them only to flag them as topics for further discussion. However, it is worth noting that including all and only those agents who are capable of undertaking an initiative does at least have the virtue of picking out a group that would, in many cases, be relatively easy to identify.

We should end this section on the moral deference model with an important clarification: the model does not rely on a commitment to any particular moral theory. Proponents of a range of different moral theories could accept norms of the sort described above, though they would assign different statuses to them.

A rule consequentialist, for example, might treat these norms as genuine moral principles—principles that determine which acts are right and which are wrong. According to one formulation of rule consequentialism, a rule of action is a genuine moral principle just in case it is part of the set of rules of action whose general acceptance can be expected to have consequences as good as the general acceptance of any alternative set of rules.^31^ Given the risk of premature or erroneous action created by the unilateralist’s curse and the likelihood that most agents are not sophisticated enough belief-formers to apply our meta-rationality model, it is plausible that the optimal set of rules will contain a norm of the sort that we have discussed.

On some other moral theories, these norms would serve not as genuine moral principles, but as guidelines for helping agents to comply with such principles. Adherents of many moral theories, both consequentialist and deontological, could accept something like the following moral principle:

Agents have moral reasons to undertake an initiative if and only if that initiative would contribute to the common good, and to spoil an initiative if and only if that initiative would detract from the common good.

Norms of the sort discussed above could help agents to better comply with this principle in unilateralist situations.^32^

## 4. Discussion

We proposed:

*The Principle of Conformity*

When acting out of concern for the common good in a unilateralist situation, reduce your likelihood of unilaterally undertaking or spoiling the initiative to a level that *ex ante* would be expected to lift the curse.

We also outlined three different ways in which agents who find themselves in unilateralist situations might comply with this principle. We do not claim that any one of these models is superior to the others in all situations. Which model should be adopted will depend, among other things, on the sophistication of the agents, the degree of communication and coordination that is possible, and the nature of existing laws and conventions bearing on the decision.

In this section we discuss a concern that might be raised regarding our principle.

Adoption of the principle of conformity is meant to make things better. Yet if we “backtest” the principle on historical experience, it is not at all clear that universal adoption of the principle of conformity would have had a net positive effect. It seems that, quite often, what is now widely recognized as important progress was instigated by the unilateral actions of mavericks, dissidents, and visionaries who undertook initiatives that most of their contemporaries would have viewed with hostility and that existing institutions sought to suppress. The benefits of iconoclasm and defiance of authority have been stated especially forcefully in the Enlightenment tradition and by proponents of scientific and technological progress. They are also evident in many cases of “whistleblowing.”

Consider the case of Daniel Ellsberg, famous for leaking the Pentagon Papers, which revealed the hopelessness of the US military situation in Vietnam. Most of Ellsberg’s peers, who had the high-level security clearance required to access the relevant documents, presumably did not believe that leaking the material to the press would contribute positively to the common good. If Ellsberg had sought to follow the principle of conformity, for example by imagining a vote among all those in a position to leak the documents, it would seem he would have had to conclude that the documents ought not be leaked. This might seem an undesirable outcome.

It is possible that the appearance that unilateralism has historically been mostly for the good is illusory. Historical unilateralism might be more salient when it worked out well than when it worked out badly, perhaps because successes have been more extreme but less frequent than the failures.

Moreover, it may be that, in some cases where the principle of conformity appears to recommend a net harmful course of action, this implication can be avoided by attending to how the group of (imaginary or actual) voters or epistemic peers is defined. For example, if one allows that these groups might be defined more broadly than the group of agents capable of undertaking an action, it may be possible to avoid the implication that Ellsberg should have refrained from whistleblowing. (Suppose that many “outsiders” would have voted in favor of his releasing the information.)

However, even if unilateralism *has* historically provided a net benefit to humanity, this need not undermine our argument. The claim that the unilateralist curse is an important phenomenon and that we have reason to lift it is consistent with the claim that the curse has provided a net benefit to humanity.

The main effect of the curse is to produce a tendency towards unilateral initiatives, and if it has historically been the case that there have been other factors that have tended to strongly inhibit unilateral initiatives, then it could be the case that the curse has had the net effect of moving the overall amount of unilateralism closer to the optimal level. For example, it might be argued that the scholars of past ages were usually far too deferential to authority, for reasons independent of the factors discussed in this paper. Their failure to take into account our arguments might then have had the salutary effect of not further inhibiting whatever propensity remained to promote new thoughts.

## 5. Concluding Thoughts

We have described a moral analog of the winner’s curse. The unilateralist’s curse arises when each of a group of agents can, regardless of the opposition of others, undertake or spoil an initiative that has significant effects on others. In such cases, if each agent decides whether to undertake (or spoil) the initiative based on his own independent naive assessment of its value, there will be a group-level bias towards undertaking (spoiling) the initiative. Importantly, this effect arises even if all the agents are assumed to be motivated solely by concern for the common good.

We proposed a principle—the principle of conformity—which instructs agents faced with a unilateralist situation to reduce their likelihood of unilaterally undertaking (or spoiling) the initiative. We then outlined three models for accomplishing this. They involved, respectively, (1) sharing information and reasoning before forming one’s evaluation of the initiative, (2) adjusting one’s evaluation in the light of the curse, and (3) deferring to the group in making one’s decision.

As we acknowledged in the previous section, there may be considerations that militate against the principle of conformity. For example, if there is already a group-level bias against unilateralism, then compliance with the principle would exacerbate this bias. However, we maintain that there is a *prima facie* case for complying with the principle. Moreover, since the level of bias due to such other factors towards or against unilateralism presumably varies across different contexts, it is likely that there will be some contexts in which the *prima facie* case for complying with the principle will be decisive. Those will be the contexts in which the group-level bias due to the unilateralist’s curse is greater than any countervailing bias against unilateralism.

It is also possible that, at least within the domain of science, the principle of conformity is more relevant today than it was, say, prior to the Enlightenment. At that time, there was, plausibly, a strong bias against thinking and acting independently in intellectual matters, at least where this would involve diverging from the views of the Church. Since the Enlightenment, however, there may have been a significant weakening of this bias. Independence of thought and action is now more widely regarded as a virtue in scientists and other intellectuals. Honors and prizes are won based on claims to originality and precedence. There may now be no bias, or only a weak bias, against unilateralism in science. Thus, the risk posed by the unilateralist curse in scientific contexts may be greater now than ever.

To resist the unilateralists’ curse one first has to become aware of when one is in a curse situation. We hope this paper will help achieve that.

## Funding

This work was supported by the The Oxford Martin School; The Wellcome Trust [grant number WT087211].

## Notes

1 We assume that the common good is determined in part by the wellbeing of all persons and other morally significant individuals. However, we remain neutral on precisely how individual wellbeing determines the common good. For example, we do not commit ourselves to the view that the common good is simply aggregate individual wellbeing; we allow that the distribution of wellbeing might be relevant. We also allow that factors besides individual wellbeing might influence the common good. For example, some initiatives might possess intrinsic value that is independent of their contribution to wellbeing, and we allow that this intrinsic value might be one element in the common good.

2 The Progressive Magazine (1979).

3 Rotblat (1985)

4 Bowden (2007).

5 Williams (1995).

6 Oye et al. (2014), Gurwitz (2014), and Oye and Esvelt (2014).

7 Thaler (1988).

8 The probability that a particular agent will be wrong about the sign of the value of the outcome is Pr(*V* ^*^ + *d* > 0) if *V* ^*^ < 0 and Pr(*V* ^*^ + *d* < 0) if *V* ^*^ > 0. This is equal to 1 − *F*(−*V* ^*^) if *V* ^*^ < 0 and *F*(−*V* ^*^) if *V* ^*^ > 0. The probability that out of *N* agents at least one will be wrong about the sign is (1 − *F*(−*V* ^*^)^*N*^) if *V* ^*^ < 0 and (1 − (1 − *F*(−*V* ^*^))^*N*^) if *V* ^*^ > 0. However, even if errors are symmetric around 0, the expected outcome is not: in the *V* ^*^ < 0 case it is enough that one agent acts for a negative value to be obtained, while in the *V* ^*^ > 0 case all agents have to err on the side of caution for them to lose out on a positive value. The expected value obtained by naive agents is hence *V* ^*^(1 − *F*(−*V* ^*^)^*N*^). For positive values this is close to *V* ^*^ (for unbiased error distributions), and we will hence focus on the *V* ^*^ < 0 case where unilateral action is a problem.

9 Theorem: As *N* grows, the likelihood *P* of at least one agent proceeding incorrectly increases monotonically towards 1 unless *F*(−*V* ^*^) = 1 (i.e. unless there is an upper limit on the size of the deviations and *V* ^*^ is more negative than this limit, no agent will ever make a sufficiently bad mistake). Proof: If *F*(−*V* ^*^) = 1, *p* = 0 for all *N*. Otherwise 0 ≤ *F*(−*V* ^*^) < 1, and hence *F*(−*V* ^*^)^*N*^ approaches 0 as *N* → ∞.

10 There will also, of course, be cases where an agent’s decision whether to undertake an initiative affects others but the agents are motivated by self-interest rather than the common good. In these cases, there are two possible reasons for getting the wrong decision, from the point of view of the common good: (i) self-interest and the common good come apart—that is, one is judged to have positive value and the other negative value—and (ii) the agent overestimates the “self-interest” value of the initiative.

11 If the distribution of errors is *skewed* such that the typical estimate is higher than the true value, for instance due to optimism bias, then the risk of erroneous action is increased: in that case, even a single agent might be likely to overestimate the value of the initiative sufficiently to undertake it even when the true expectation value of the initiative is strongly negative. But this is unrelated to the curse. In the case of estimates skewed towards safety—that is, where there is pessimism bias—any tail distribution allowing mistaken action will still produce a growing probability of going ahead as *N* grows. There may be intermediary cases where the curse would helpfully serve to balance out an opposite effect arising from pessimism bias.

12 Condorcet (1785).

13 Cf. Snorri Sturluson’s *Gylfagining*.

14 For a list of UN Security Council vetoes, see http://research.un.org/en/docs/sc/quick (accessed February 23, 2015).

15 See, for example, MacFarquhar (2011).

16 In addition to adhering to the principle of conformity in particular unilateralist situations, one might also have some moral reason to work at a more general level to counteract the unilateralist’s curse. One way to do this would be to promote awareness and adoption of the principle of conformity. Another way would be to promote the development of institutions that make unilateralist situations less likely to arise, especially in regards to matters of global significance where the effects of the curse can be particularly devastating.

17 Bostrom (2011).

18 Aumann (1976).

19 For discussion, see e.g. Christensen (2009) and Feldman and Warfield (2010).

20 Attempts to weaken this assumption have been made; see Hanson (2006).

21 Cox and Isaac (1984).

22 In actual cases, the other agents are likely to have different priors and non-independent information, plus uncertainty about the number of agents. This possibility can be included in our top equation, at the price of a far more complex model that needs several priors.

23 Including self-deception about how meta-rational they are (Cowen and Hanson 2001).

24 Another way of looking at the problem is through the lens of game theory. Each agent needs to choose a (pure or mixed) strategy mapping their observations into actions, trying to maximize expected utility. We assume that all agents share a single utility function, i.e. they are all working for the common good. Since the agents know they are identical and will not be able to communicate, they will be using the same strategy. It can then be shown that there if there is any local maximum in their utility function if they all use the same strategy *g*, then the general use of *g* is a Nash equilibrium. (See Armstrong (2012) for further details.) The equilibrium can be non-strict under some conditions: a single agent is free to follow a different strategy without changing the outcome. This means that no agent will be able to realize higher expected value pursuing a different strategy. Note that optimal strategies can be probabilistic (i.e. mixed). For example, suppose the information each agent received is either a red light or a green light (indicating whether the initiative should be undertaken), but the green light is only correct 75% of the time. For multiple agents, always undertaking the initiative when a green light is received produces a worse outcome than only acting on a green light with a probability less than one. As the number of agents goes up this probability should become lower, exploiting the fact that in the case the action does have positive outcome the likelihood of at least one agent acting remains high enough. Calculating the optimal probability requires an estimate of the number of agents and the probability of erroneous information, again requiring Bayesian priors. Game theory mainly tells us that a solution exists, but finding it requires the meta-rationality approach.

25 The norm does not deal with “spoiler” cases, where one agent can prevent an initiative from taking place. However, an analogous norm could be adopted to lift the unilateralist curse in those cases.

26 Jackson et al. (2001) and Cello, Paul, and Wimmer (2002).

27 See Atlas et al. (2003) and Journal Editors and Authors Group (2003). This procedure was invoked in the wake of two recent studies which demonstrated how to make avian influenza transmissible by air between ferrets. See, for discussion, Perez (2012), Faden and Karron (2012) and Osterholm and Henderson (2012).

28 See, for a brief description of the case, Berg (2008).

29 The assumptions of the Condorcet theorem can be weakened in many ways. In particular, agent competence only has to be on average above 50% (Grofman, Owen, and Feld 1983), and a certain level of voting correlation does not reduce majority voting performance (Ladha 1992).

30 For similar reasons, an analogous norm would tend to reduce the likelihood of *spoiling* an initiative of those who evaluate an initiative most negatively.

31 See, for example, Hooker (2002).

32 A parallel can be drawn to one prominent justification for the authority of the law, due to Joseph Raz. That justification appeals to the same kind of consideration that we suggest could ground a norm against unilateral action: “The normal and primary way to establish that a person should be acknowledged to have authority over another person involves showing that the alleged subject is likely better to comply with reasons which apply to him (other than the alleged authoritative directives) if he accepts the directives of the alleged authority as authoritatively binding, and tries to follow them, than if he tries to follow the reasons which apply to him directly” (Raz 1994, 214).

33 In this case, the maximum likelihood estimate is simply the average of their individual estimates.
